# The Interaction Effects of Suicidal Ideation and Childhood Abuse on Brain Structure and Function in Major Depressive Disorder Patients

**DOI:** 10.1155/2021/7088856

**Published:** 2021-07-21

**Authors:** Wei Wang, Lijun Kang, Nan Zhang, Xin Guo, Peilin Wang, Xiaofen Zong, Lihua Yao, Hanping Bai, Jing Cheng, Ning Tu, Hongyan Feng, Gaohua Wang, Lihong Bu, Fei Wang, Zhongchun Liu

**Affiliations:** ^1^Department of Psychiatry, Renmin Hospital of Wuhan University, Wuhan, Hubei, China; ^2^PET/CT/MRI and Molecular Imaging Center, Renmin Hospital of Wuhan University, Wuhan, Hubei, China; ^3^Early Intervention Unit, Department of Psychiatry, Affiliated Nanjing Brain Hospital, Nanjing Medical University, Nanjing, China; ^4^Functional Brain Imaging Institute of Nanjing Medical University, Nanjing, China

## Abstract

Suicidal ideation (SI) is a direct risk factor for suicide in patients with depression. Regarding the emergence of SI, previous studies have discovered many risk factors, including childhood abuse as the major public problem. Previous imaging studies have demonstrated that SI or childhood abuse has effects on brain structure and function, respectively, but the interaction effects between them have not been fully studied. To explore the interaction effect between SI and childhood abuse, 215 patients with major depressive disorder completed the Childhood Trauma Questionnaire to evaluate childhood abuse and Beck's Scale for Suicidal Ideation to evaluate SI. Then, they completed magnetic resonance imaging (MRI) within one week after completing questionnaires. Respectively, we preprocessed the structural and functional images and analyzed gray matter volumes (GMV) and mean fractional amplitude of low-frequency fluctuation (mfALFF) values. Results showed that the changes of GMV in the cuneus, precuneus, paracentric lobule, inferior frontal gyrus, and caudate nucleus and local activity in cuneal and middle temporal gyrus are in relation with SI and childhood abuse. And in left caudate, SI and childhood abuse interact with each other on the influence of GMV. That is, the influence of SI in GMV was related to childhood abuse, and the influence of childhood abuse in GMV was also related to SI. Therefore, the combination of SI and childhood abuse based on imaging should help us better understand the suicide ideation developing mechanism and propose more effective targeted prevention strategies for suicide prevention.

## 1. Introduction

According to the published statistics, up to 90% of those who commit suicide may have mental disorders. In addition, 50–70% of them may suffer from the major depressive disorder (MDD) [1]. Repeatedly thinking about death or self-injury and suicide prompts severe depression. Past studies have shown that persistent SI is a high-risk factor leading to suicide [2]. SI is affected by several other factors, such as age, gender, the severity of depression, impairment of social function, and family history of suicide [3, 4]. Meanwhile, past studies have reported that childhood abuse is significantly associated with an increased risk of SI. There is a greater likelihood for a person to think about suicide if he or she had suffered from severe trauma in childhood [5]. Childhood abuse includes physical and emotional abuse, neglect, and sexual abuse before the age of 16 years, which often leads to the development of serious consequences, including not only an increased risk of SI but also a huge socioeconomic burden [6–8]. The Interpersonal–Psychological Theory of Suicide believes that childhood abuse is a risk factor for SI in adulthood [9]. Angst et al. and Björkenstam et al. also confirmed this view from their research [10, 11]. Therefore, childhood abuse can be considered to be a predictor of SI [12]. Another study of patients with depression demonstrated that patients with SI scored significantly high on emotional abuse and neglect [13]. Smith et al. [14] suggested that childhood abuse can be a powerful predictor of SI because the trauma caused by all forms of childhood abuse is associated with the lack of a sense of belonging and responsibility. At present, most studies conducted across the world focus on the impact of a single type of childhood abuse, but only a few studies have assessed the various forms of childhood abuse [15].

Previous studies have reported that the biological basis of SI in patients with MDD involves changes in the brain structure and function [16]. One clinical study has revealed that MDD patients with SI possess different functional collections in the middle frontal gyrus compared to MDD patients without SI [17, 18]. The middle frontal gyrus is involved in the acquired ability of suicide networks in men [19]. Past studies based on voxel-based morphometry (VBM) have shown that people with SI have a decreased cortical volume in the left middle frontal gyrus relative to that in healthy people [20]. Therefore, the changes in the middle frontal gyrus are believed to be an important biological marker of SI [16]. Various psychological abnormalities associated with the development of suicide indicate potential interference in the fields of cognition, execution, inhibition, and emotion. The two key brain regions responsible for processing emotional and cognitive information, especially emotional stimulation and executive function, are the amygdala and the prefrontal cortex [21, 22]. Another study reported that, when compared with healthy controls and MDD patients without SI, the gray matter volume (GMV) of MDD patients with SI was decreased in the left and right dorsolateral prefrontal cortex and in the right ventral prefrontal cortex, which further adds to the supportive evidence reported by Wang et al.'s study [23, 24]. In addition, the posterior cingulate cortex and the parahippocampal region can be considered to be interactive interfaces for emotion, cognitive assessment, and memory [25–29]. In clinical cases, reduced GMV of the frontoparietal cerebellar network was recorded in depressed patients with SI as well as decreased executive function, cognitive inflexibility, and impaired decision-making and problem-solving abilities [30–36].

Some other studies have reported that childhood abuse is associated with abnormal brain structure and function. Marshall et al. [37] found that exposure to childhood abuse can have a negative impact on brain development, often increasing the risk for the development of psychopathological symptoms. A meta-analysis based mainly on adult participants revealed that abuse is linked to reduced GMV in the prefrontal cortex and ventral superior temporal gyrus [38]. Another meta-analysis reported differences in GMV of the amygdala, but not in the hippocampus region. It was also reported that adults previously exposed to childhood abuse displayed an increase in the size of the right amygdala when compared with other adults without such an experience [39]. Overall, the most consistent findings were concentrated in the ventromedial and dorsal prefrontal cortex as well as the lateral temporal lobe cortex [40, 41]. The decrease in the cortical thickness in these areas may be related to the various forms of interruption of emotional regulation [42]. In a study based on functional magnetic resonance imaging (fMRI), the activation of the dorsolateral and dorsomedial prefrontal cortex was observed with an increase in abused adolescents during cognitive reassessment when compared with that in nonabused adolescents [43]. It can thus be inferred that childhood abuse is associated with structural and functional changes in the lateral and ventromedial frontal lobes, which may lead to behavioral and emotional control issues [44].

In the past, concerns and changes related to suicide prevention did not effectively reduce the suicide rates [45]. Identifying the risk factors and protective factors that can better predict the risk of suicide is of critical significance. Because SI occurs before a person makes suicidal attempts, identifying SI is essential to prevent the risk of suicide [46]. Meanwhile, childhood abuse is believed to be a risk factor for SI. Therefore, the combination of childhood abuse and SI based on imaging is expected to facilitate the comprehension of the mechanism of SI development and propose better-targeted successful prevention strategies for suicide prevention. We thus hypothesized that the development of SI and childhood abuse is related to the changes in the structure and function of certain brain areas, involving interaction effects between SI and childhood abuse in certain brain areas.

## 2. Methods and Materials

### 2.1. Participants and Design

All patients included in this study visited the outpatient clinic of the Renmin Hospital of Wuhan University from July 2020 to January 2021. Two experienced psychiatrists diagnosed the MDD patients based on the DSM-5 criteria. After their enrollment, the patients were explained about the study in detail and their consent was obtained. The MDD patients who signed the informed consent forms were included in the “Early warning system and comprehensive intervention for depression” (ESCID), a website employed to enroll patients with depression and to evaluate the severity of their presenting symptoms. The exclusion criteria included the following: (1) psychiatric diseases, except MDD, diagnosed according to the DSM-5; (2) history of severe head trauma or intracranial disease; (3) severe stiffness or other symptoms that could interfere with the study; (4) transcranial magnetic stimulation (TMS) or MECT treatment within 6 months; (5) pregnancy; and (6) being left-handed. Next, the patients filled the basic information in the questionnaire and underwent the following tests: Digit Symbol Substitution Test (DSST) [47], Childhood Trauma Questionnaire (CTQ) [48], and Beck's Scale for Suicidal Ideation (BSS) [49], and completed MRI within 1 week.

All the patients participating in our study were categorized into 2 groups according to their BSS test results: MDD patients without SI (MDD) and MDD patients with SI (MDD-SI). Similarly, the groups MDD1, MDD2, MDD3, MDD4, MDD5, and MDD6 were created, which included MDD patients without any childhood abuse, without emotional abuse, without physical abuse, without sexual abuse, without emotional neglect, or physical neglect, respectively. The groups of MDD-CTQ, MDD-EA, MDD-PA, MDD-SA, MDD-EN, and MDD-PN included MDD patients with at least one type of childhood abuse, emotional abuse, physical abuse, sexual abuse, emotional neglect, and physical neglect, respectively.

This study protocol was approved by the Ethics Committee of Renmin Hospital of Wuhan University, Wuhan, Hubei, China.

### 2.2. Research Instruments

#### 2.2.1. General Information Questionnaire

The general information questionnaire asked for demographic data such as gender, age, somatic diseases, and past diagnosis and treatment.

#### 2.2.2. DSST

The subjects were asked to fill the corresponding symbols in order within 90 s. The final score reflected the subjects' processing speed, executive functions, learning abilities, memory capacity, and attention capacity [47].

#### 2.2.3. CTQ

A questionnaire is designed to evaluate the experience of individuals before the age of 16 years concerning emotional abuse, physical abuse, sexual abuse, emotional neglect, and physical neglect. When the value of emotional abuse ≥ 13 or physical abuse ≥ 10 or sexual abuse ≥ 8 or emotional neglect ≥ 15 or physical neglect ≥ 10, the patient was considered to have a history of childhood abuse. When the above criteria were not met, the patient was considered to have no history of childhood abuse [48].

#### 2.2.4. BSS

Beck et al. compiled this scale in 1979 to quantify and evaluate SI. This scale is divided into 2 parts; the first 5 questions were used to determine the presence of SI and the last 14 questions to assess the severity of SI. When the answers to questions 4 and 5 were “no,” we believed that the patient had no SI within nearly 1 week. Otherwise, the patient was believed to have SI and was expected to complete the next 14 questions [49].

### 2.3. MRI Acquisition

MRI data was acquired at the PET center of Renmin Hospital of Wuhan University using a 3.0 T scanner (General Electric, Milwaukee, USA). Spin echo-planar imaging (EPI) sequence was used in structural imaging, with the following parameters: repetition time (TR) = 8.5 ms, echo time (TE) = 3.2 ms, preparation time = 450 ms, flip angle (FA) = 120, visual field (FOV) = 256 mm, acquisition matrix = 256 mm, slice thickness = 1 mm, slice gap = 0 mm, and locs per slab = 180. The scanning time was 4 minutes and 41 seconds. Resting-state fMRI requires subjects to be quiet, close their eyes, breathe smoothly, in a more comfortable position, without any physical movement, and do not carry out any thinking activities. EPI sequence was used, axial scanning was performed for 212 times, 32 slices, slice thickness = 3.0 mm, slice gap = 0 mm, interval = 1 mm, repetition time (TR) = 2000 ms, echo time (TE) = 30 ms, flip angle (FA) = 90°, acquisition matrix = 64 × 64, and visual field (FOV) = 240 × 240 m^3^. The scanning time was 16 minutes.

### 2.4. Data Processing

The structural imaging data were preprocessed based on the VBM8 toolbox (http://dbm.neuro.uni-jena.de/vbm8/) in Statistical Parametric Mapping 8 (SPM 8; https://www.fil.ion.ucl.ac.uk/spm/software/) to perform data conversion, test quality, segment and normalize, extract index, retest the quality, and smooth. The original imaging data collected were in the DICOM format and required conversion into the NIFTI format for processing. The purpose of segment and normalization was to separate the gray matter, white matter, and cerebrospinal fluid and to ensure that the images of all subjects were in the same space, and the anatomical positions corresponding to the same coordinates were consistent. The normalization process was conducted by the Diffeomorphic Anatomical Registration Through Exponentiated Lie algebra (DARTEL) algorithm to the Montreal Neurological Institute (MNI) template. We then extracted the GMV of all the subjects. All structural images were smoothed with an 8 mm full-width at the half-maximum (FWHM) Gaussian filter.

The Restplus V1.2 toolbox in SPM 12 was used to preprocess the resting-state fMRI data. After data conversion, the first 10 volumes were discarded to reach the steady state. In addition, we conducted slice timing to complete the time-level correction. The spatial-level correction includes realignment and normalization. The subjects with excessive head movement (>3 mm or >30) according to the realignment parameter were excluded. Normalization was performed using the DARTEL algorithm to the MNI template. All functional images were smoothed with a 6 mm full-width at a half-maximum (FWHM) Gaussian filter. Then, we performed detrend, nuisance covariate regression, and filtering (0.01–0.08 Hz). The values of mALFF could be extracted using the above processes.

The abovementioned operations were conducted in the MATLAB R2013b platform (MathWorks, Sherborn, MA, USA).

### 2.5. Statistical Analyses

The difference in the gender and the results of CTQ between the MDD group and the MDD-SI group was calculated by Chi-square analysis. The Mann–Whitney *U*-test was applied to measure the differences in age between the 2 groups. The difference between the 2 groups regarding the DSST results was explored by 2 independent sample *t*-test. The abovementioned analysis was completed using the IBM SPSS Statistics (Version 26.0). The analysis of GMV and mfALFF was executed in SPM 12 by a two-sample *t*-test and full factorial. Post hoc analysis of the region of interest (ROI) was conducted in Restplus V1.2 based on the MATLAB R2013b and IBM SPSS Statistics (Version 26.0) by analysis of variance (ANOVA) and pairwise comparison. Imaging findings were considered to be significant at *P* < 0.001, corrected by the Gaussian random field (GRF) correction, while the other findings were considered to be significant at *P* < 0.05.

## 3. Results

### 3.1. Differences in Demographics and Clinical Characteristics

A total of 215 patients were enrolled in the study, of which 18 did not complete the BSS questionnaire, 2 did not complete the CTQ questionnaire, and 30 could not undergo MRI due to scheduling issues. Of the 185 patients who underwent MRI, 16 showed obvious abnormalities in the brain structure, such as the transparent septum, and 19 failed to obtain their fMRI images. The reasons for the same included the patient's inability to complete the entire examination process and the loss of data during data transfer. According to the BSS results, there were 44 individuals in the MDD group (22.34%) and 153 in the MDD-SI group (77.66%). According to the CTQ outcomes, there were 130 (61.03%) participants who had experienced abuse in their childhood and 83 (38.97%) who had not experienced any type of abuse. Specifically, 55 (28.21%) individuals experienced emotional abuse, 37 (18.97%) physical abuse, 29 (14.87%) sexual abuse, 95 (48.72%) emotional neglect, and 84 (43.08%) physical neglect. The results revealed no significant difference with respect to age, gender distribution, head motion, and exposure rates of various types of childhood abuse cases (*P* > 0.05) between the MDD group and the MDD-SI group. The proportion of SI in first-onset patients was higher (*χ*^2^ = 4.316, *P* = 0.038). When compared with patients with low scores, those with high HAMD-17 scores included a higher proportion of patients with SI (*Z* = −4.530, *P* < 0.001). In addition, in patients with SI, the DSST score was lower (*t* = 2.531, *P* = 0.013) (Table 1).

### 3.2. Differences in GMV

**Figure 1:**
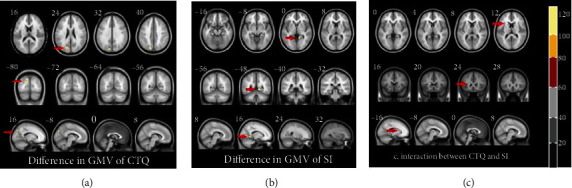
Differences in GMV. (a) Difference in GMV between MDD1 and MDD-CTQ in left cuneus. (b) Difference in GMV between MDD and MDD-SI in right lingual. (c) Interaction between CTQ and SI in left caudate.

**Figure 2:**
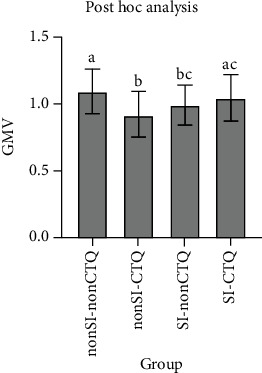
GMV of ROI extracted according to interaction effect. Notes: nonSI-nonCTQ: MDD patients without suicidal ideation and childhood abuse; nonSI-CTQ: MDD patients without suicidal ideation but with childhood abuse; SI-nonCTQ: MDD patients with suicidal ideation but without childhood abuse; SI-CTQ: MDD patients with suicidal ideation and childhood abuse.

We noted differences in GMV among the groups MDD1 and MDD-CTQ, MDD5 and MDD-EN, and MDD6 and MDD-PN in the left cuneus (*T* = −3.899, *P* < 0.001; *T* = −4.053, *P* < 0.001; *T* = −3.536, *P* < 0.001). In the left paracentral lobule, the GMV of the MDD-PA group was significantly larger than that of the MDD3 group (*T* = −3.955, *P* < 0.001). In terms of MDD patients with sexual abuse, the GMV of MDD patients without sexual abuse was larger in the left triangular portion of the left inferior frontal gyrus (*T* = 4.1578, *P* < 0.001). Moreover, a difference of GMV was also noted in the left precuneus between the MDD5 and MDD-EN groups (*T* = 4.558, *P* < 0.001). The GMV of patients with SI was significantly smaller than that of patients without SI in the right lingual gyrus (*T* = 3.777, *P* < 0.001). The results of full factorial between SI and childhood abuse suggested differences in the left caudate (*T* = 13.589, *P* < 0.001). Post hoc analysis revealed that, in the left caudate, the GMV of nonSI-nonCTQ was larger than that of nonSI-CTQ (*I* − *J* = 0.173, *P* = 0.003) and SI-nonCTQ (*I* − *J* = 0.104, *P* = 0.040), while that of nonSI-CTQ was smaller than that of SI-CTQ (*I* − *J* = −0.122, *P* = 0.006) (Tables 2 and 3; Figures 1 and 2).

### 3.3. Differences in mfALFF

The mfALFF value of the MDD-PA group was significantly lower than that of the MDD3 group in the left cuneus (*T* = 4.514, *P* < 0.001), while that of the MDD-SA group was lower than that of the MDD4 group in the left middle temporal gyrus (*T* = 4.238, *P* < 0.001). However, there were no significant results based on the full factorial experiment (*P* > 0.001; Table 4).

## 4. Discussion

Our demographic analyses revealed no significant difference in the distribution of sex, age, head motion, and incidence of childhood abuse between the MDD and MDD-SI groups. As such, our results conform to those of previous studies partially. A clinical study revealed that age was not significantly associated with SI within the past 4 weeks, although it was negatively correlated with SI within the past 1 year; however, there was no significant correlation with respect the gender and SI [50]. Another study by Eswatini revealed that women of age 25–34 years were more likely to develop SI [51]. Some past studies have also suggested that the impact of gender on SI is related to puberty, as this gender difference was not observed in prepubertal youth; on the other hand, the incidence of SI in women (15.7%) was higher than that in men (12.4%) after puberty [52]. However, with regard to the incidence of childhood abuse, our results were not completely consistent with those of previous studies. A large-sized clinical study on pregnant women revealed that pregnant women with a history of childhood abuse had a high risk of developing SI, especially when they had been subjected to emotional abuse, physical abuse, and sexual abuse [53]. A meta-analysis reported that childhood abuse was associated with an increase in SI occurrence, but a higher heterogeneity was reported by only a few studies [54]. The difference in the inferences reported by different studies may be related to the difference in the cultural backgrounds, economic development levels, sample size, and research standards across the studies.

The DSST score of the MDD patients with SI was significantly lower than that of MDD patients without SI. This observation was generally consistent with that reported by previous studies, many of which suggested that SI is associated with neurocognitive impairment, especially inattention, memory loss, and executive function, such as response inhibition and impaired decision-making [55–58]. Therefore, the early identification of defects of attention, memory, and executive functions may provide an opportunity for early intervention to prevent SI occurrence. People with SI respond to real events in a desperate cognitive schema, with the belief that the difficulties encountered will not be resolved in the future and will not tolerate pain [50]. In other words, cognitive intervention is of great significance toward reducing SI. Specific interventions in cognition such as attention, impulse, problem-solving, and decision-making can maximize the advantages of existing SI-intervention methods [59].

The results of the VBM-based study revealed that the GMV in the left cuneus of MDD patients with prior experience of childhood abuse was larger than that of those who had not experienced any childhood abuse. Previous studies have also indicated that childhood abuse can lead to changes in the brain structure, although the specific structural changes recorded vary from a study to another. Past studies have also shown that childhood abuse is associated with decreased GMV in the hippocampus, corpus callosum, and prefrontal cortex [60–62]. Other scholars believe that different types of abuses may have common neurobiological consequences and that the affected children may feel reduced pain because of the weakening of the development of the sensory system and pathways that transmit disgust and traumatic experiences [63, 64]. However, the analysis of the interaction effects between SI and childhood abuse based on VBM revealed that the influence of SI on the GMV of left caudate changes with whether there is childhood abuse or not. Previous studies have also demonstrated that the caudate nucleus is related to reward-processing and decision-making abilities, while childhood abuse and suicide are related to reward processing alone, which is consistent with our present results to a certain extent [65–67]. From the perspective of imaging, this finding indicates an evident correlation between childhood abuse and SI. This finding also reminds us that taking effective measures to reduce the incidence of childhood abuse may reduce the overall risk of suicide in the future.

Although there was no significant difference in the brain structure between MDD patients with emotional abuse and those without that experience, we noted that GMV in the left cuneus and precuneus of MDD patients with emotional neglect was significantly lower than that in MDD patients without emotional neglect. The results of the fMRI-based study showed that the local activity in the left cuneus of MDD patients with the experience of physical abuse was lower than that of MDD patients without this experience. The precuneus is a part of the default mode network (DMN) that participates in the processing of introspection and emotions [68]. The precuneus plays an important role in visuospatial imagery, episodic memory retrieval, and self-processing operations [69]. Previous studies have revealed that the structural and functional changes of the cuneus and precuneus are related to the differences in memory-related metacognitive abilities among different individuals [70]. This effect was also confirmed through a noninvasive low-frequency TMS study conducted in the precuneus [71]. When compared with MDD patients without the experience of physical abuse and physical neglect, those MDD patients with these experiences had greater GMV in the paracentric lobule and cuneus, respectively. The paracentric lobule is related to the movement and sensation of the lower limbs. At present, no direct relationship between this brain region and childhood abuse has been reported, although it may provide a new direction for further research. A large sample-sized study conducted in the community suggested the most significant reduction in the GMV of the right medial frontal gyrus in individuals with early exposure to severe corporal punishment [72]. Past studies on the brain regions mentioned earlier have reported that these areas are involved in addiction, suicide-related behavior (include SI), depression, and posttraumatic stress disorder (PTSD) [73–75]. However, these studies did not determine whether the observed differences in the GMV of the brain regions can be attributed to the cause or consequence of physical abuse.

The MDD patients with sexual abuse showed smaller GMV in the triangular portion of the left inferior frontal gyrus than in MDD patients without sexual abuse. The function of the prefrontal cortex is related to cognitive, emotional, pain, and behavioral management. Meanwhile, when compared with MDD patients without sexual abuse, patients with sexual abuse demonstrated lower local activity in the middle temporal gyrus. However, the results of previous studies are not completely consistent with our present studies. This difference can be possibly attributed to the fact that the decrease in the GMV of the abovementioned brain regions may precede the existence of sexual abuse and may exist as a risk factor. However, there exists no favorable evidence to support this conjecture [76].

The GMV in the lingual gyrus of MDD patients without SI was significantly higher than in those with SI, albeit no significant results were noted in their respective fMRI. Past studies support that changes in the brain structure and function are associated with an increased risk of SI, although there exist some inconsistent results for these specific areas with changes [77]. The lingual gyrus is mainly responsible for vision, especially with the processing of letters, and may be involved in logical analysis and visual memory processing. A clinical study revealed that MDD patients without SI have stronger functional connectivity in the lingual gyrus than MDD patients with SI [78]. Although our results are consistent with the findings of some past studies, there exists no evidence strong enough to support the results. However, our result suggests that the lingual gyrus demands more attention, which should be covered in future studies on SI. The results of another ROI-based study are partly consistent with these previous studies, in that the GMV in the left dorsolateral prefrontal cortex of MDD patients with SI is smaller than that of patients without SI [23]. Several past studies have suggested that the dorsal striatum plays a unique role in reflecting SI and have emphasized the importance of imaging methods to detect SI in adolescents [79]. Although the imaging changes in MDD patients with SI are not particularly clear, further technological developments and studies should be able to provide a more convenient and accurate method for the evaluation of SI.

## 5. Conclusion

MDD patients with SI have reduced GMV in the lingual gyrus, while the GMV and mfALFF value of patients who had experienced childhood abuse in the cuneus, precuneus, paracentral lobule, and inferior frontal gyrus also changed. In MDD patients, the influence of SI on the GMV of the caudate varies with whether there is childhood abuse or not. These findings cumulatively reflect on the association between childhood abuse and SI from the perspective of imaging. However, further research is warranted to determine the biomarkers that produce SI as well as to ascertain the complete pathway connecting childhood abuse with SI.

## 6. Limitation

First and foremost, compared with previous imaging studies, our sample size is sufficient, but due to the large incidence of SI and small incidence of various types of childhood abuse, the proportion of the case group and the control group is not perfectly balanced, and the sample size can be expanded in further study. Second, because the clinical study involves the changes of the patient's condition, we ask only for no TMS and MECT treatment in the past 6 months but do not limit their use of drugs. So, the effects of drugs cannot be ruled out. Third, the patients' SI and childhood abuse are evaluated by the self-rating scale, and there may be deviations when recalling, which is inevitable. Fourth, although we rule out other mental disorders that meet the DSM-5 criteria, MDD patients are often accompanied by other symptoms, such as anxiety, obsessive-compulsive, and other symptoms. It is impossible to completely rule out all these. Fifth, our study is conducted only in patients with MDD, so our results are only applicable to patients with MDD and cannot be extended to the community population.

## Figures and Tables

**Table 1:** Differences in demographics and clinical characteristics.

		MDD		MDD-SI		*χ* ^2^/*Z*/*t*	*P*
		*n*	%	*n*	%		
Gender	Female	30	20.83	114	79.17	0.696	0.404
	Male	14	26.42	39	73.58		
First episode	No	25	29.41	60	70.59	4.316	0.038^∗^
	Yes	19	16.96	93	83.04		
Head motion (>3 mm or > 3 degree)	No	27	22.13	95	77.87	0.461	0.734^a^
	Yes	2	14.29	12	85.71		
CTQ	No	20	25.97	57	74.03	1.139	0.286
	Yes	23	19.49	95	80.51		
Emotional abuse	No	34	24.29	106	75.71	1.442	0.230
	Yes	9	16.36	46	83.64		
Physical abuse	No	34	21.52	124	78.48	0.137	0.711
	Yes	9	24.32	28	75.68		
Sexual abuse	No	39	23.49	127	76.51	1.352	0.245
	Yes	4	13.79	25	86.21		
Emotional neglect	No	25	25.0	75	75.00	1.038	0.308
	Yes	18	18.95	77	81.05		
Physical neglect	No	27	24.32	84	75.68	0.775	0.379
	Yes	16	19.05	68	80.95		
Age (median) (*P*_25_ ~ *P*_75_)		23 (22~31)		23 (21~26)		-1.791^b^	0.073
HAMD-17 (median) (*P*_25_ ~ *P*_75_)		14 (7~20)		21 (16~25)		-4.530^b^	<0.001^∗^
DSST (mean ± SD)		69.67 ± 2.42		60.42 ± 1.68		2.531^c^	0.013^∗^

^∗^ *P* < 0.05 means significant difference. ^a^Fisher's exact test; ^b^*Z* score of age; ^c^*t* score of DSST.

**Table 2:** Differences in GMV.

Group 1 : Group 2	Region	Voxel	MNI coordinates			*T*/*F* values
			*X*	*Y*	*Z*	
MDD1 : MDD-CTQ	Cuneus (L)	407	-10.5	-79.5	39.0	-3.8990
MDD3 : MDD-PA	Paracentral lobule (L)	534	-9.0	-37.5	66.0	-3.9553
MDD4 : MDD-SA	Frontal-Inf-Tri (L)	642	-46.5	28.5	7.5	4.1578
MDD5 : MDD-EN	Cuneus (L)	201	-10.5	-79.5	28.5	-4.0525
	Precuneus (L)	628	-4.5	-52.5	52.5	4.5582
MDD6 : MDD-PN	Cuneus (L)	225	-18.0	-70.5	22.5	-3.5359
MDD : MDD-SI	Lingual (R)	184	13.5	-48.0	-3.0	3.7768
SI∗CTQ^a^	Caudate (L)	77	-16.5	21.0	10.5	13.5885

^a^Interaction effect of SI and childhood abuse. Notes: MDD patients without any kind of childhood abuse (MDD1); MDD patients with any kind of childhood abuse (MDD-CTQ); MDD patients without physical abuse (MDD3); MDD patients with physical abuse (MDD-PA); MDD patients without sexual abuse (MDD4); MDD patients with sexual abuse (MDD-SA); MDD patients without emotional neglect (MDD5); MDD patients with emotional neglect (MDD-EN); MDD patients without physical neglect (MDD6); MDD patients with physical neglect (MDD-PN); MDD patients without suicidal ideation (MDD); MDD patients with suicidal ideation (MDD-SI).

**Table 3:** Post hoc analysis of interaction between SI and childhood abuse.

	*I* − *J*	*P*	CI (95%)
nonSI-nonCTQ vs. nonSI-CTQ	0.173	0.003^∗^	0.0579~0.2876
nonSI-nonCTQ vs. SI-nonCTQ	0.104	0.040^∗^	0.0051~0.2039
nonSI-nonCTQ vs. SI-CTQ	0.050	0.286	-0.0424~0.1429
nonSI-CTQ vs. SI-nonCTQ	-0.068	0.150	-0.1615~0.0250
nonSI-CTQ vs. SI-CTQ	-0.122	0.006^∗^	-0.2085~-0.0365
SI-nonCTQ vs. SI-CTQ	-0.054	0.096	-0.1182~0.0098

^∗^ *P* < 0.05 means significant difference.

**Table 4:** Differences in mfALFF.

Group 1 : Group 2	Region	Voxel	MNI coordinates			*T* values
			*X*	*Y*	*Z*	
MDD3 : MDD-PA	Cuneus (L)	132	12.0	-90.0	24.0	4.5144
MDD4 : MDD-SA	Temporal-Mid (L)	39	-48.0	-60.0	0	4.2377

Notes: MDD patients without physical abuse (MDD3); MDD patients with physical abuse (MDD-PA); MDD patients without sexual abuse (MDD4); MDD patients with sexual abuse (MDD-SA).

## Data Availability

The data used in this study to support our findings are questionnaires and DICOM statistics, and they are available from the corresponding authors on reasonable request.
